# Research on electromagnetic characteristics of traction motor rotary shafts with variable parameter defects based on finite element method

**DOI:** 10.1038/s41598-023-36455-z

**Published:** 2023-06-21

**Authors:** Mengmeng Song, Mengwei Li, Xiaoyan Song, Shungen Xiao, Feng Jiang, Naiqiu Huang

**Affiliations:** 1 College of Information and Mechanical & Electrical Engineering, Ningde Normal University, Ningde, China; 2 College of Mechanical and Electrical Engineering, Fujian Agriculture and Forestry University, Fuzhou, China; 3 School of Automation, Wuxi University, Wuxi, China

**Keywords:** Mechanical engineering, Applied physics

## Abstract

In view of the breakage of the rotary shaft of the traction motor of the locomotive, in order to ensure the reliability and safety of the equipment, this paper studies the magnetic field containing rich information in the space around the rotary shaft, so that the crack defect can be detected and the crack size can be judged in time. In order to study the influence of defect geometric parameters (width, depth and hidden depth) on the spatial magnetic field distribution around the defect in the process of eddy current testing of metal shafts, based on the principle of eddy current testing, a metal rotary shaft detection model with different defect parameters was established by COMSOL software for simulation. The horizontal magnetic induction intensity, vertical magnetic induction intensity and their respective phases are used to analyze the magnetic field distribution around the defect under different parameters. The results show that the magnetic field related parameters can qualitatively and quantitatively evaluate the crack width, qualitatively evaluate the relative size of the crack depth, and qualitatively judge whether there are hidden cracks.

## Introduction

In practical engineering applications, mechanical equipment generally carries a variety of complex loads, and the various environments in which they continue to work are relatively harsh, including humidity, high pressure, and high temperature. The mechanical properties of these equipment will gradually decrease after a long time of work, and even the equipment will fail. The reason is that in the equipment there are crack defects and corrosion. Non-destructive testing (NDT) technology is significant because it can find these defects in time, which is conducive to ensuring the product quality of these equipment^1–3^. The basis of NDT technology is modern science and technology, which is a comprehensive subject. It is usually used to judge the internal or surface structure, physical properties and state parameters of the tested part. The judgment is usually based on changes in parameters such as sound, light, and magnetism caused by electromagnetic fields, and usually will not destroy the measured object^4^. At present, the commonly used methods of NDT in engineering are mainly the following, such as magnetic particle testing (MT), ultrasonic testing (UT), eddy current testing (ECT), radiographic testing (RT) and penetration testing (PT). Among them, one of the most commonly used NDT techniques is ECT, because of its many characteristics, such as low requirements on the testing surface, no need for contact, fast testing speed, no need for coupling, easy operation and no radiation to the human body. It plays an important part in some aspects because of these advantages, such as testing and evaluating quality and structural integrity, and the detection objects are mainly metal materials, parts and equipment^5–7^.

As one of the NDT methods, the ECT technology is based on the principle of electromagnetic induction. It uses the magnetic field (MF) energy coupling between the measured object and the probe coil to detect the measured object. This technology is very suitable for testing the integrity of the test piece^8^. In terms of experimental research on the electromagnetic properties of defects, the measurement of the induced current-related MF usually uses solid-state sensors, such as Hall sensors, magnetometers, superconducting quantum interference devices (SQUID), giant magnetoresistance (GMR) sensors, etc.^9–12^. In order to quantitatively evaluate the austenitic stainless steel, Jongwoo et al.^13^ obtained the electromagnetic field distribution near the tested part by using the Hall sensor array. A new detection method was proposed by Kim, which is used to detect the cracks in the riveting part of the aircraft. This method uses the GMR sensor to collect the response signal^14^. Espina-Hernández^15^ conducted related experiments using GMR sensors to discover the relationship between output voltage and crack size. It was found that the peak voltage difference of the output signal of the magnetic field sensor is affected by the width and depth of the crack, and the peak position of the output signal is proportional to the crack width. Liu et al.^16^ collected the MF changes near the cracks of aluminum alloy materials by GMR sensors, and found that it is feasible to detect cracks. Jiang et al.^17^ accurately inverts the shape and position information of the defect by using SQUID to measure the magnetic signal around the defect. In order to study the change of the MF when the defect and the scanning direction are different, Guo et al.^18^ used the finite element software to carry out simulation modeling and data analysis. Through the analysis, it was found that the peak-to-peak distance of the signal increases with the increase of the defect span. The amount of change in length will make the amount of change in the MF signal consistent with it. Because the MF is disturbed by defects, the distribution will change, and Zhang et al.^19^ analyzed it by the finite element method. Analyzing the results, it can be found that the maximum value of the normal MF (located at the first and last ends of the crack) can be used to quantitatively determine the length of the crack, and the depth has a certain corresponding relationship with the minimum amplitude of the tangential MF. This relationship can be used to quantitatively calibrate the depth. Tian of Newcastle University in the UK proposed to use characteristic information such as zero crossing point, peak value, time rising point and peak time to evaluate material defects^20–23^. Zhou^24^ used the Hall sensor to obtain the pulse signal, and the output peak and peak time characteristics of the defect can be extracted from it to determine the depth of the crack. Jiang^25^ proposed a method to quantitatively evaluate cylindrical corrosion defects by using the lateral influence nodes and longitudinal depression depth in the butterfly diagram. An eddy-current magneto-optical imaging system was proposed by Elshafiey and Fitzpatrick. This system is based on the Faraday rotation effect and can be well used to detect corrosion and cracks near the surface^26,27^. Magneto-optical imaging technology has the advantages of reliability and speed, which have been proved by many experiments, so large-area fatigue cracks and corrosion such as aircraft skins are very suitable for detection by this technology^28–30^. Gao et al.^31^ made an ultrasonic comparison test block according to the actual flaw detection requirements, and realized the flaw detection of the locomotive shaft by using the specific waveform for damage. Su et al.^32^ and others realized the identification and re-inspection of the defects of the rotating shaft by exploring the magnetic marks in the magnetic particle inspection of the rotating shaft. Liu et al.^33^ realized the detection of a malignant defect in the shaft of the traction motor through ultrasonic flaw detection. Chen et al.^34^ introduced the finite element sub-model method for hairline solid modeling analysis, verified the effectiveness of using the finite element sub-model for hairline simulation, and provided effective technical support for the evaluation and acceptance of traction motor shaft hairline. Yu et al.^35^ selected high-performance flaw detectors and probes to improve the signal-to-noise ratio of the eddy current flaw detector, and realized the eddy current inspection without dismantling the axle box and without removing the paint. However, some deficiencies of traditional ECT began to arise, because modern testing technology has been improving, and the requirements for testing quality are constantly increasing. These deficiencies include: non-destructive evaluation is the future development trend. This method can only detect and judge the presence or absence of defects. It can no longer meet the current needs; it is impossible to realize accurate positioning of defects; smaller defects often go undetected. Therefore, studying and analyzing the electromagnetic field distribution around tiny defects is very important, so as to determine whether there is a quantitative relationship between the spatial MF around the defect and its geometric parameters. In this regard, this paper selects the traction motor shaft commonly used in the field of transportation as the research object. A locomotive or motor car is generally pulled by a traction motor through the interference fit between the rotating shaft cone and the pinion. The rotating shaft bears both bending moment and torque during the operation of the locomotive. Bending stress and torsional shearing stress are generated inside the shaft at the same time. An important component for transmitting torque, the shaft of the traction motor is the key bearing part of the locomotive drive mechanism, and its working condition directly affects the safety of train operation. Therefore, the identification and quantitative evaluation of shaft defects are extremely important. Timely detection of defects and intelligent fault diagnosis can screen out unqualified products, thereby ensuring equipment safety and reliability^36^. In this paper, the COMSOL finite element simulation software is used to construct the defect detection model with different geometric parameters, and the relationship between the MF and the defect is analyzed by observing the horizontal magnetic induction intensity (abbreviated as HMII), vertical magnetic induction intensity (abbreviated as VMII) and their respective phases (abbreviated as PHMII and PVMII) of the spatial MF near the defect.

## Eddy current testing theory

### The principle of ECT

**Figure 1:**
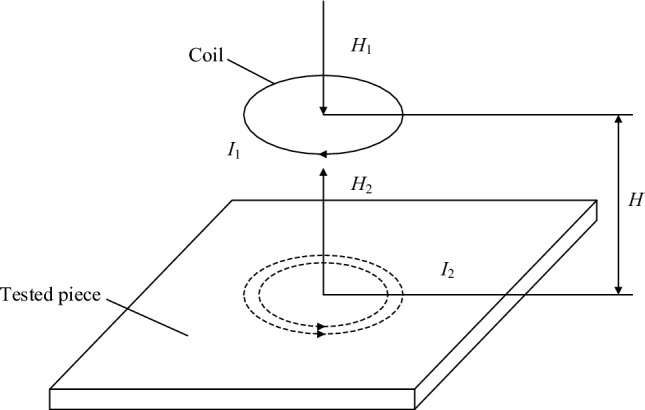
Schematic diagram of eddy current testing.

One important application of the eddy current (EC) effect is ECT. The principle of ECT is shown in Fig. 1. When an alternating current is applied to the coil, an original alternating magnetic field is generated around the coil. The original alternating magnetic field acts on the specimen, which will induce eddy currents. The induced eddy current will excite the induced magnetic field, which hinders the original magnetic field. By measuring the coil impedance, the detection of specimen defects can be realized.

The application object of ECT technology is conductive materials. Usually, the bulk density of free charges of metal conductive materials is set to 0, because the movement time of free charges is very short. At this time, the Maxwell equations can be written as:

1 $$\nabla \times {\text{H }} = (\sigma + {\text{j}}\omega \varepsilon ){\text{E}}$$

2 $$\nabla \times E = - j\omega \mu H$$

3 $$\nabla \cdot H = {0}$$

4 $$\nabla \cdot E = {0}$$

Among them, taking the curl of formula (1) and substituting it into formula (2), we can get:

5 $$\nabla \times \nabla \times H = (\sigma + j\omega \varepsilon )\nabla \times E$$

According to the vector relationship $\nabla \times \nabla \times P = \nabla (\nabla \cdot P) - \nabla^{{2}} P$, and in formula (3)$\nabla \cdot H = {0}$, then we get:

6 $$- \nabla^{{2}} H = (\sigma + j\omega \varepsilon )\nabla \times E$$

Substituting formula (2) into formula (6), we get:

7 $$\nabla^{{2}} H - (j\omega \mu \sigma - \omega^{{2}} \mu \varepsilon )H = {0}$$

From this, it can be found that the motion form of the electromagnetic field in the medium is a wave. In the actual calculation, it is found that the value of the first term in the brackets of formula (7) is much larger than the value of the second term. This is because the electrical conductivity of the metal is about 10^7^ Ω^−1^ m^−1^, and the vacuum dielectric constant *ε*_0_ = 8.85 × 10^–12^ F/m, at this time the ratio of σ in the first item to *ωε* in the second item is about 10^–9^, so the second item can be ignored directly, then formula (7) can be simplified as:

8 $$\nabla^{{2}} H - j\omega \mu \sigma H = {0}$$

Similarly, we can also get:

9 $$\nabla^{{2}} E - j\omega \mu \sigma E = {0}$$

10 $$\nabla^{{2}} J - j\omega \mu \sigma J = {0}$$

Equations (8) to (10) are called electromagnetic penetration equations, which are used to explore the propagation of electromagnetic energy in conductive metals, and are also theoretical equations for ECT technology. The meanings of the physical quantities mentioned above are: $\nabla \times$ is the curl operator; *H* is the magnetic field strength in A/m; *σ* is the conductivity in Ω^−1^ m^−1^; *ε* is the dielectric constant in F/m; *E* is the electric field strength in C/m^2^; *μ* is the magnetic permeability in H/m; $\nabla \cdot$ is the divergence operator; *J* is the current density in A/m^2^.

### The impedance analysis method

**Figure 2:**
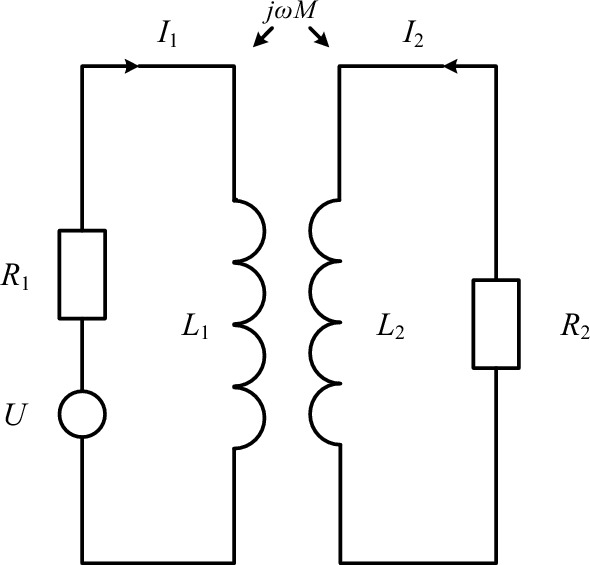
Equivalent circuit diagram.

At present, the impedance analysis method is widely used in eddy current testing, and the equivalent circuit diagram of its coil coupling is shown in Fig. 2.

In Fig. 2 R1 and R2 represent the resistance of the probe coil and the tested piece respectively; *L*_1_ and *L*_2_ are the inductances of the probe coil and the tested piece respectively; *M* is the mutual inductance between the probe coil and the tested piece; *U* is the excitation voltage at both ends of the probe coil.

According to Kirchhoff's voltage law, the voltage equations in the primary and secondary circuits are:

11 $$\left\{ \begin{gathered} (R_{1} + j\omega L_{1} )I_{1} - j\omega MI_{2} = U; \hfill \\ (R_{2} + j\omega L_{2} )I_{2} - j\omega MI_{1} = 0. \hfill \\ \end{gathered} \right.$$

Simultaneously solving the equations in (11), the equivalent impedance of the probe coil can be obtained as:

12 $$Z = \frac{U}{{I_{1} }} = R_{1} + \frac{{(2\pi f)^{2} M^{2} }}{{R_{2}^{2} + (2\pi f)^{2} L_{2}^{2} }}R^{2} + j[2\pi fL_{1} - \frac{{(2\pi f)^{3} M^{2} L^{2} }}{{R_{2}^{2} + (2\pi f)^{2} L_{2}^{2} }}]$$

By further solving, the equivalent resistance of the real part and the equivalent inductance of the imaginary part of the coil can be obtained as follows:

13 $$\left\{ \begin{gathered} R = R_{1} + \frac{{(2\pi f)^{2} M^{2} }}{{R_{2}^{2} + (2\pi f)^{2} L_{2}^{2} }}R_{2} ; \hfill \\ L = 2\pi fL_{1} - \frac{{(2\pi f)^{3} M^{2} L_{2} }}{{R_{2}^{2} + (2\pi f)^{2} L_{2}^{2} }}. \hfill \\ \end{gathered} \right.$$

where the equivalent resistance *R* is a function of the mutual inductance coefficient *M*. It can be observed that *M* increases due to the decrease of the distance between the probe and the tested object, which has nothing to do with whether the tested object is a magnetic material or not. Two effects will affect the equivalent inductance *L*: the magnetostatic effect affects *L*_1_, that is, whether the magnetic material of the test piece is related to *L*_1_; the eddy current effect affects *L*_2_, and the equivalent inductance is oppositely affected by the two effects. Therefore, when the soft magnetic material is used as the tested object, the static magnetostatic effect mainly affects the equivalent inductance in the coil. When the probe is close to the tested object, the equivalent inductance of the probe increases; When non-ferromagnetic material or hard magnetic material is used as the tested object, the eddy current effect mainly affects the equivalent inductance in the coil, and the equivalent inductance of the probe decreases.

### Skin effect

In the related problems of ECT, the attenuated magnetic field induces EC, which will cause the attenuation of the EC inside the conductor specimen. This phenomenon is called the skin effect, that is, the current decays with the increase of depth, and the surface current of the conductor specimen visibly focused. The penetration depth refers to the distance that the EC penetrates into the conductor. The penetration depth when the EC density decays to 1/e (about 36.8%) of its surface value is defined as the standard penetration depth, also called the skin depth. The formula for calculating the penetration depth of EC is^37,38^:

14 $$\delta = \frac{1}{{\sqrt {\pi \sigma \mu } }}\frac{1}{\sqrt f }$$

where *δ* is the penetration depth in mm; *f* is the frequency of AC current in Hz; *μ* is the magnetic permeability of the conductor in H/m; *σ* is the conductivity of the conductor in S/m.

## Finite element model of eddy current inspection with variable parameters for defect detection

### Geometric modeling

**Figure 3:**
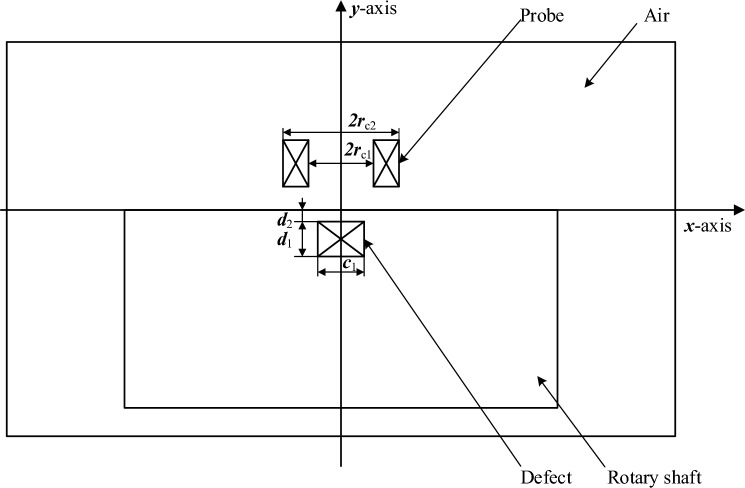
Equivalent model of variable parameter defect detection system (at defect axis section).

For conductors and other lossy materials that generate a large amount of induced current in a time-varying magnetic field, the AC/DC module in COMSOL can be used to model, and the equivalent model in Fig. 3 can be established. Among them, *c*_1_ is the width of the crack, *d*_1_ is the depth of the crack, *d*_2_ is the hidden depth of the crack,* r*_c1_ is the inner diameter of the coil, which is set to 2 mm, and *r*_c2_ is the outer diameter of the coil, which is set to 4 mm. The built model contains two types of boundary conditions: the strong boundary condition is the first type of boundary condition, which refers to the periphery of the plane area in the figure, which represents the range of the solution area. The second type of boundary condition is the natural boundary condition, which is the boundary strip between various media in the model. As long as the boundary conditions are set to change continuously, the extreme value of the functional function will automatically satisfy it.

### Material selection

Select air as the material of the air field, select copper as the coil material, select stainless steel as the material of the tested piece. The relative magnetic permeability *μ*_r_, electrical conductivity *σ* and relative permittivity *ɛ*_r_ of the selected materials are shown in Table 1.

**Table 1:** Material parameters.

Material	*μ*_r_	*σ*/(S m^−1^)	*ɛ*_r_
Air	1	0	1
Copper	1	5.998 × 10^7^	1
Stainless steel	100	1.137 × 10^6^	1

### Addition of physics

There are many modules in COMSOL, here we select the “Low Frequency Electromagnetic Fields” module as needed as the physical field of this model. Set up the “Uniform Multi-Turn Coil” in the magnetic field so that the number of turns of the coil and the applied current can be determined. Choose 500 turns as the number of turns of the coil, choose the current signal of 0.05A as the excitation, and apply the Dirichlet boundary condition (that is, the magnetic vector potential is zero) to the coil model of the eddy current probe.

### Mesh generation

**Figure 4:**
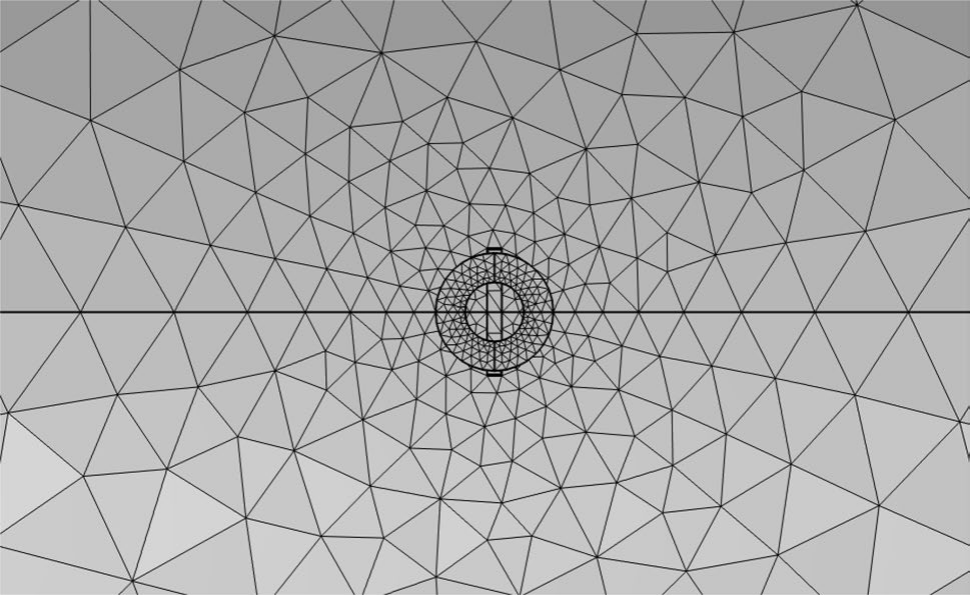
Mesh division diagram of the model (vertical view).

**Figure 5:**
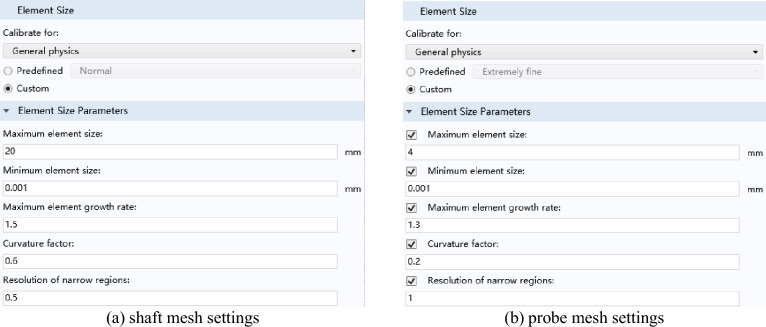
Shaft and probe mesh settings.

In the time-varying electromagnetic field, the skin effect will appear in the rotating shaft conductor specimen. At the same time, the magnetic field distribution of defects and probe coil accessories is the key content of this research. Under the premise of not affecting the accuracy of the results, a free triangular mesh is used for the air domain and the probe coil, and extremely fine is selected as the mesh size. Figure 4 shows the mesh division the model. Both the eddy current probe and the rotating shaft adopt self-adaptive grids. Since the probe is relatively precise, the grid division is extremely fine, and the volume of the rotating shaft is relatively large, so conventional division can be used. Their specific grid settings are shown in Fig. 5a,b as shown.

### Solving and post-processing of finite element model

Enter the excitation frequency into the model and set the correct solution for the model. Solving the situation where the defects have different geometric parameters in the model can obtain the magnetic field distribution of the corresponding situation, and then analyze the relationship between the magnetic field and the geometric parameters of the defect (width, depth and hidden depth), the electromagnetic properties near the defect can be obtained, and their influence on the electromagnetic properties near the defect can also be studied by setting different frequencies, coil turns, and excitation current amplitudes.

### Magnetic cloud image near the crack

**Figure 6:**
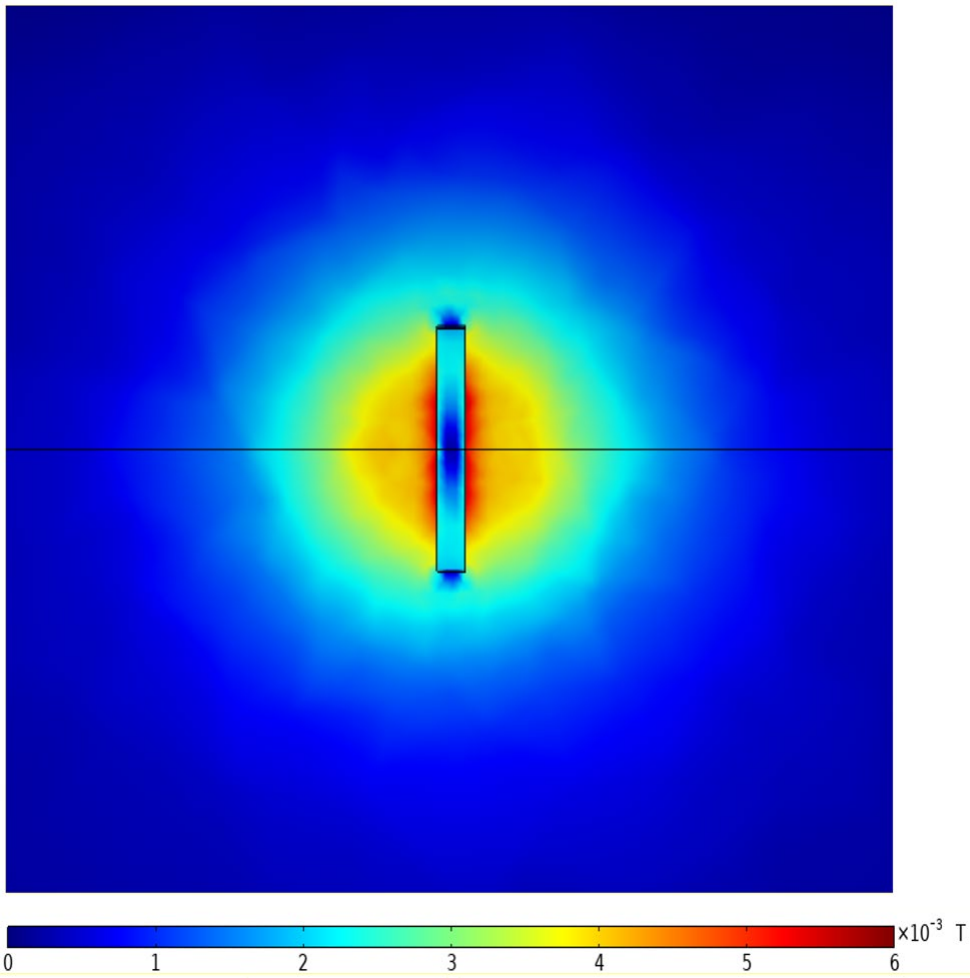
Top view of the distribution of magnetic induction intensity on the surface of the rotating shaft.

After the solution is completed, the change of the magnetic induction intensity in the horizontal direction can be analyzed first, and the top view can be selected for observation. Taking the case where the width and depth of the crack are both 1 mm as an example, the distribution of the magnetic induction intensity near the defect in the rotating shaft is shown in Fig. 6. It can be seen from Fig. 6 that the magnetic induction intensity is small at the crack and edge position. As the distance from the crack center increases, the magnetic induction increases first and then decreases.

**Figure 7:**
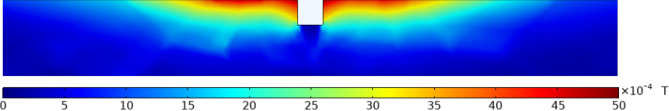
Front view of magnetic induction intensity distribution on the surface of the rotating shaft.

Select a three-dimensional cross-section perpendicular to the horizontal plane, and take this cross-section as an observation plane, on which the change of magnetic induction intensity can be drawn, and the front view shown in Fig. 7 can be obtained. It can be seen from Fig. 7 that with the increase of the distance from the surface, the magnetic induction intensity gradually decreases.

## Results and discussion

### Magnetic field distribution law of crack defect with different widths

Keep the crack depth *d*_1_ = 0.5 mm and the hidden depth *d*_2_ = 0 mm unchanged, and study its influence on the MF around the defect by changing the crack width. The influence of different crack widths on the distribution of the MF is mainly manifested in the amplitude of HMII and VMII and their respective phases, the range of crack width is *c*_1_ = 0.01 mm (considered to be in a non-defective state), *c*_1_ = 1 mm, *c*_1_ = 2 mm, *c*_1_ = 4 mm, *c*_1_ = 6 mm, *c*_1_ = 12 mm.

**Figure 8:**
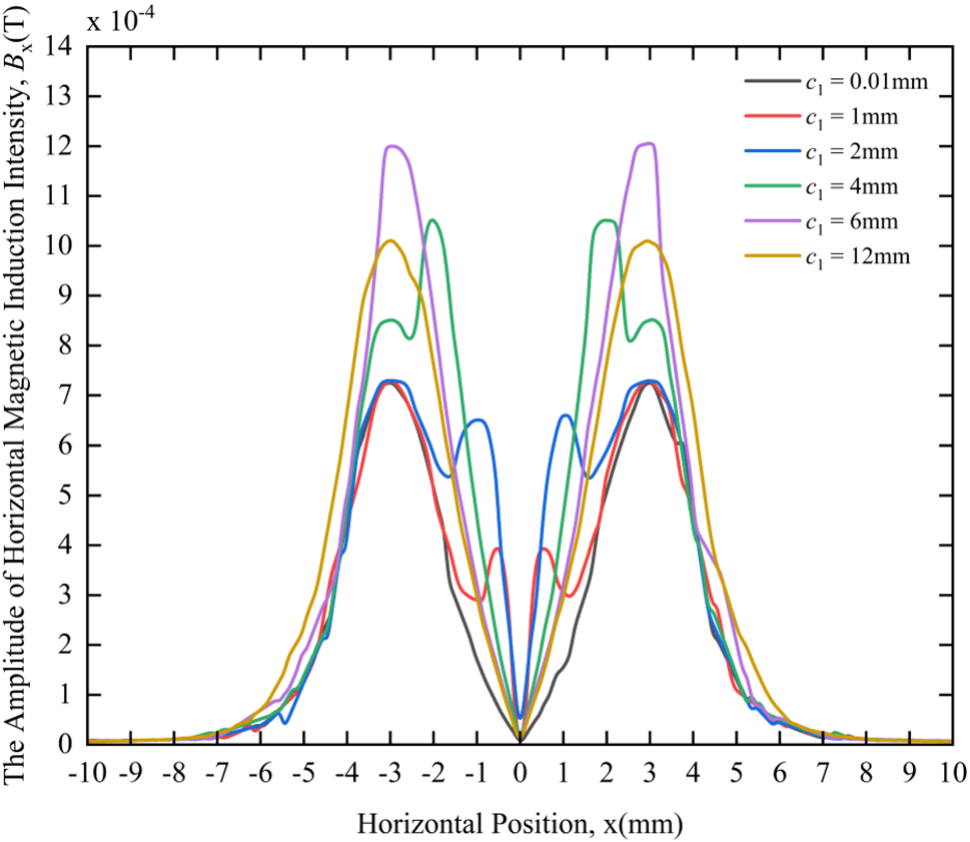
Variation curves of the HMII under different crack widths.

Figure 8 shows the variation law of the HMII amplitude under different crack widths. From the magnetic field responses of different crack widths in Fig. 8, following rules can be obtained:

1. Under different crack widths, the distribution of HMII is very different. When the crack widths are 1 mm, 2 mm, 4 mm, 6 mm, and 12 mm, the variation curves of the horizontal component *B*_x_ of the magnetic induction intensity around the cracks are obviously different from the case where the crack width is 0.01 mm (considered no defect), that is, the change curve has a pair of symmetrical peaks or the original peak value becomes larger.
2. When *c*_1_ = 0.01 mm, as the horizontal distance from the 0 coordinate increases, the HMII gradually increases. It reaches the max value at the center of the coil((*r*_c1_ + *r*_c2_)/2 = 3 mm, define this value as the equivalent radius of the coil), then decreases slowly, and gradually tends to zero.
3. When *c*_1_ = 1 mm, 2 mm and 4 mm, there will be two pairs of peaks in the variation curve of HMII. The distance between a pair of peaks close to the 0 coordinate is equal to the crack width *c*_1_, and the peak value of the pair of peaks gradually increases when the crack width gradually increases; the distance between the pair of wave peaks that is slightly farther from the 0 coordinate is equal to twice the equivalent radius of the coil, which is 6 mm, and the peak value of the pair of wave peaks remains basically unchanged when the crack width is different.
4. When *c*_1_ = 6 mm and 12 mm, that is, when the crack width is equal to or greater than 2 times the equivalent radius of the coil, the HMII curve only appears a pair of peaks that are symmetrical about the 0 coordinate, and the peak positions appear at − 3 mm and 3 mm respectively, which is similar to the case of *c*_1_ = 0.01 mm, but when the crack width is 6 mm or 12 mm, the peak value of the two peaks is higher than that of 0.01 mm.

As shown in Fig. 8, a pair of peaks close to the 0 coordinate is defined as the characteristic peaks to distinguish them from the original peaks at − 3 mm and 3 mm, and the distance between the two peaks of the characteristic peaks is defined as the characteristic peak distance. From the above phenomena, it can be seen that in the range of 0 < *c*_1_ < 6 mm, the influence range of the surface MF gradually increases with the increase of the crack width, because the distance between the peak-to-peak values of the characteristic wave peaks gradually increases. When *c*_1_ ≥ 6 mm, with the increase of the crack width, the influence range of the MF is basically unchanged, only showing that the peak value of the original peak increases, and the characteristic peak no longer appears. Therefore, the width of the crack can be quantitatively analyzed through the characteristic quantity of the characteristic peak distance, and the size of the characteristic peak distance reflects the size of the crack width.

By comparing with the change curves of HMII in the non-defect state, it can be qualitatively judged whether the crack exists, and whether the crack width is greater or less than 6 mm can be deduced by whether there is a characteristic peak. As shown in Fig. 8, when the crack width is less than 6 mm, the characteristic peak will appear in the HMII variation curve, but when the crack width is greater than 6 mm, the HMII variation curve will not appear the characteristic peak. Although the characteristic peak will not appear, the peak value of the original peak will increase, which can be used to distinguish whether there is a crack.

**Figure 9:**
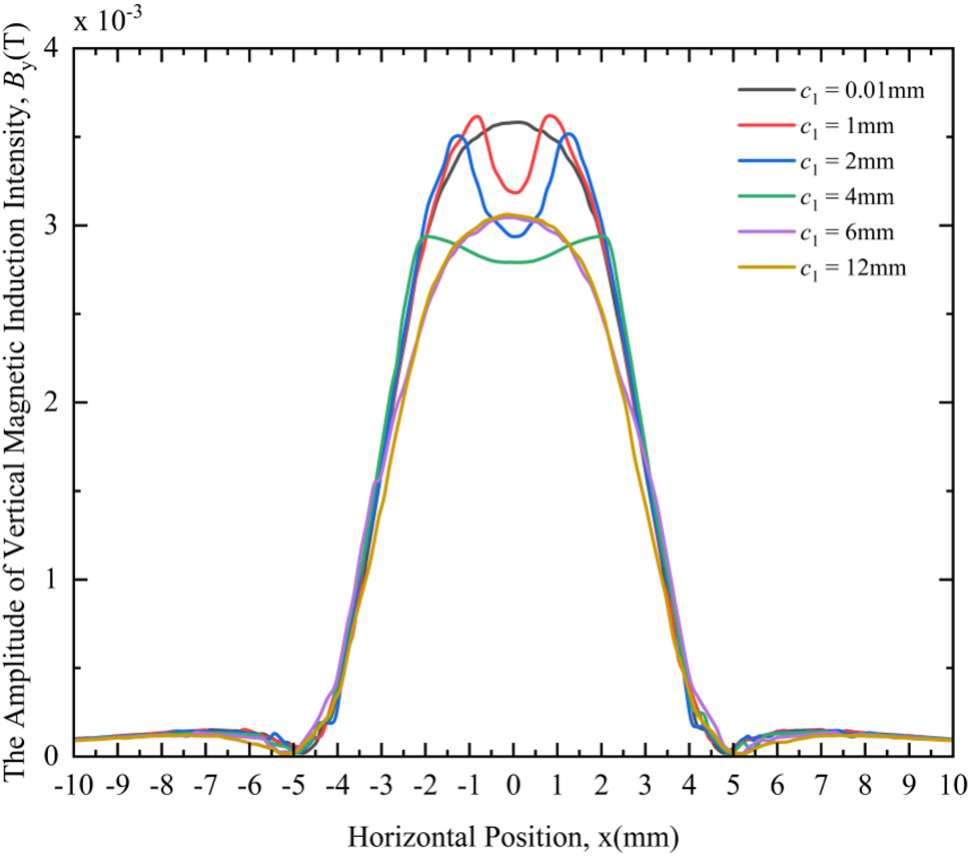
Variation curves of the VMII under different crack widths.

Figure 9 shows the variation law of the VMII amplitude under different crack widths. From the MF responses of different crack widths in Fig. 9, the following rules can be obtained:

1. Under different crack widths, the distribution of VMII is very different. When the crack widths are 1 mm, 2 mm, 4 mm, 6 mm, and 12 mm, the variation curves of the vertical component By of the magnetic induction intensity around the cracks are obviously different from the case where the crack width is 0.01 mm (considered no defect), that is, the change curve has a depression at the 0 coordinate or the peak value of the original wave peak decreases.
2. When *c*_1_ = 0.01 mm, with the increase of the horizontal distance from the 0 coordinate, the VMII gradually decreases and tends to zero, then increases slightly and then decreases again, and the overall appearance is "low on both sides, high in the middle". A peak appears at the 0 coordinate.
3. When *c*_1_ = 1 mm, 2 mm and 4 mm, the VMII variation curve will have a depression at the 0 coordinate, and as the crack width gradually increases, the depression here becomes deeper and deeper.
4. When *c*_1_ = 6 mm and 12 mm, the VMII change curve has the same basic trend as that of the non-defect state, but the peak value at the 0 coordinate will drop significantly, and the change curves in these two states are basically the same, that is, when *c*_1_ ≥ 6 mm, as the crack width gradually increases, the VMII variation curve remains basically unchanged.

From the above phenomena, we can qualitatively judge whether the crack exists by comparing it with the VMII change curve in the non-defect state. If there is a depression at the 0 coordinate of the change curve or the peak value at this point drops significantly, the crack exists, otherwise the crack does not exist. Moreover, it can be deduced whether the crack width is less than 6 mm or greater than 6 mm according to whether the change curve is depressed at the 0 coordinate or the peak value is significantly decreased. As shown in Fig. 9, when the crack width is less than 6 mm, the VMII change curve will have a depression at the 0 coordinate; and when the crack width is greater than 6 mm, the peak value at the 0 coordinate of the magnetic induction intensity change curve will drop significantly.

**Figure 10:**
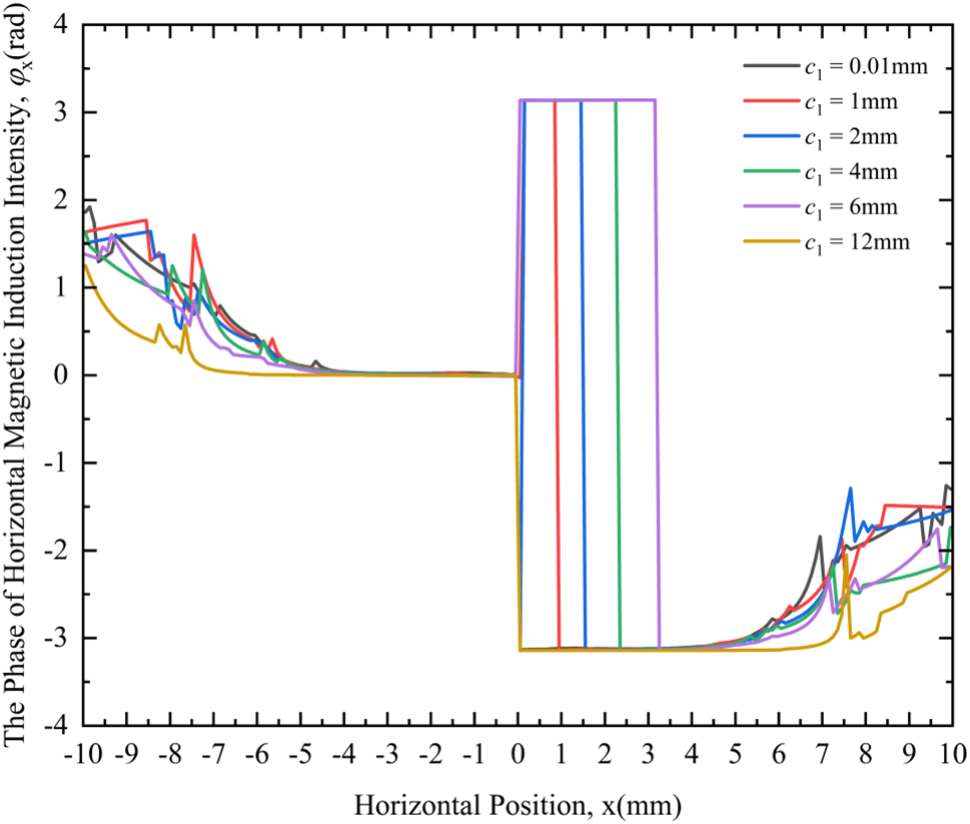
Variation curves of the PHMII under different crack widths.

Figure 10 shows the variation law of the PHMII under different crack widths. From the MF responses of different crack widths in Fig. 10, the following rules can be obtained:

1. Under different crack widths, the PHMII is very different. When the crack width is 1 mm, 2 mm, 4 mm, 6 mm respectively, the change curve of the phase *φ*_x_ of the HMII around the crack is obviously different from the case where the crack width is 0.01 mm (considered no defect), that is, there will be a period of time where the phase remains at π.
2. With the gradual increase of the crack width, the phase lag of the HMII gradually increases, and the phase interval of π expands. When the crack width is 12 mm, the phase lag phenomenon of the HMII disappears, and there is no longer an interval where the phase remains at π, and the change curve is basically consistent with the defect without cracks.

It can be seen from the above phenomena that the phase information of the HMII can be used to detect the crack width. When the crack defect is not very large, it can be qualitatively judged whether the crack defect exists by comparing it with the phase change curve of the HMII in the state without defects. If there is a phase lag, there is a defect; otherwise, there is no defect. Moreover, the crack width can be deduced qualitatively by comparing the size of the interval whose phase is maintained at π. For the defect with a large crack width, since the crack defect is beyond the main influence range of the coil, its existence cannot be inferred from the phase information of the HMII.

**Figure 11:**
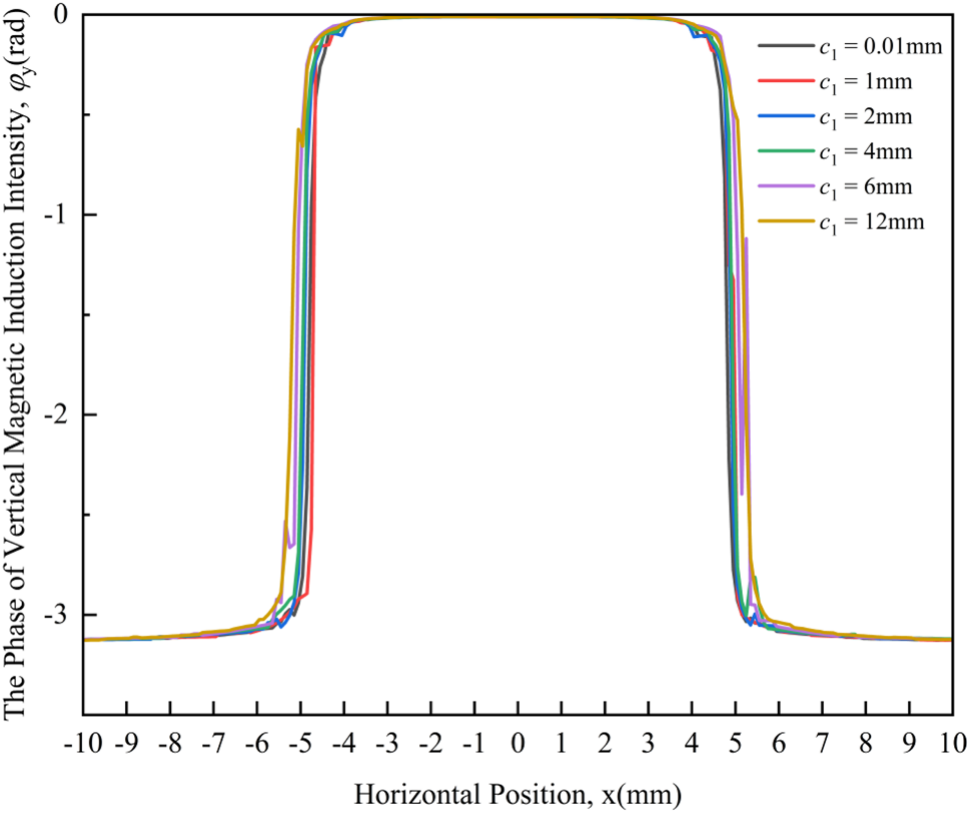
Variation curves of the PVMII under different crack widths.

Figure 11 shows the change law of the PVMII under different crack widths. It can be seen from the figure that under different crack widths, the phase change curves of the VMII have little difference and are difficult to distinguish. This shows that the PVMII is not sensitive to the crack width, so the PVMII cannot be used as the characteristic quantity of crack width detection.

In this section, whether there are characteristic peaks at different positions on the horizontal magnetic induction variation curve, or the sag in the center of the vertical magnetic induction variation curve makes symmetrical peaks appear at different positions, the reason is that the magnetic permeability of the air gap at the cracks of different widths is significantly smaller than the relative magnetic permeability of the rotating shaft, a part of the magnetic field will bypass the crack and pass through the nearby objects, and the magnetic field lines will concentrate on both sides of the crack, resulting in a sharp increase in the magnetic induction intensity here. The hysteresis phenomenon of the horizontal magnetic induction intensity change curve is ultimately due to the change of the relative permeability of the region caused by the crack, which leads to the distortion of the magnetic force line, and the magnetic force line flows to the area with smaller reluctance on both sides.

### Magnetic field distribution law of crack defect with different depths

Keep the crack width *c*_1_ = 2 mm and the hidden depth *d*_2_ = 0 mm unchanged, and study its influence on the surface MF around the defect by changing the crack depth. The influence of different crack widths on the distribution of the MF is mainly manifested in the amplitudes of the HMII and VMII and their respective phases. Among them, the variation range of the crack depth is *d*_1_ = 0.01 mm (considered a non-defective state), *d*_1_ = 0.5 mm, *d*_1_ = 1 mm, *d*_1_ = 2 mm, *d*_1_ = 3 mm, *d*_1_ = 6 mm.

**Figure 12:**
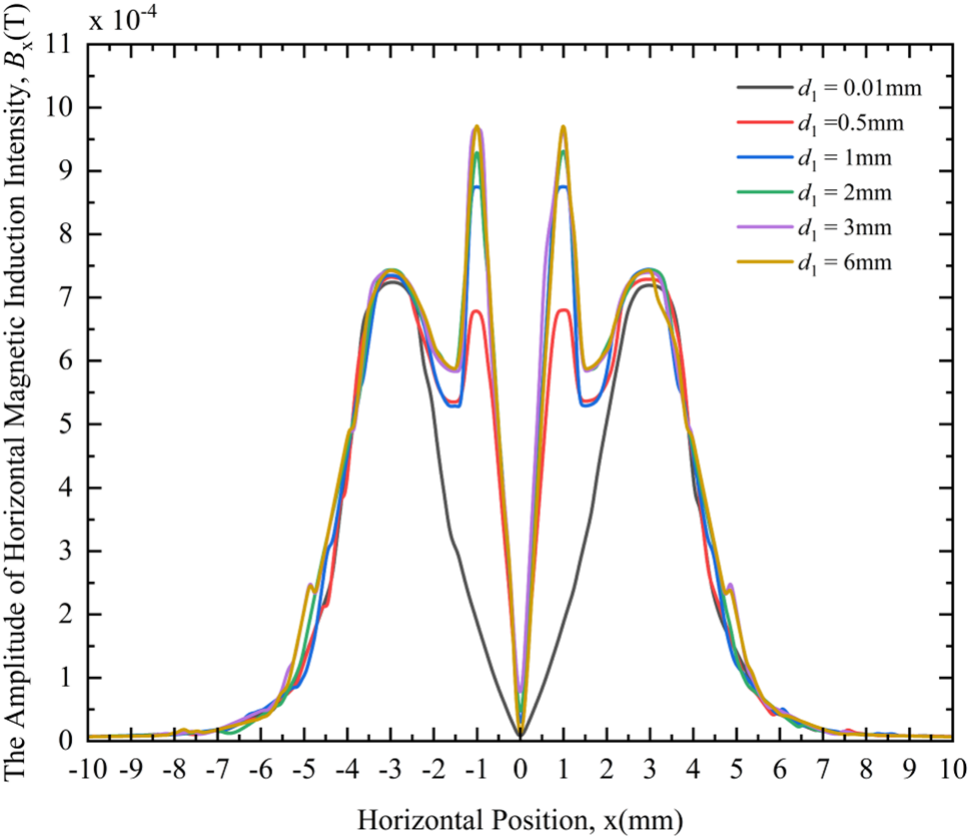
Variation curves of the HMII under different crack depths.

Figure 12 shows the change law of the amplitude of the HMII under different crack depths. From the MF responses of different crack depths in Fig. 12, the following rules can be obtained:

1. At different crack depths, the distribution of HMII is very different. When the crack depths are 0.5 mm, 1 mm, 2 mm, 3 mm and 6 mm, the change curve of the HMII around the crack is obviously different from the case where the crack depth is 0.01 mm (considered no defect), and the change curve will have a pair of symmetrical peaks, which are still defined as the characteristic peaks here.
2. Except for the crack depth of 0.01 mm, the peak value of the characteristic wave peak gradually increases with the increase of the crack depth, and when the crack depth increases to a certain extent, the peak value of the characteristic wave peak begins to stabilize and basically no longer increases.
3. When the crack depth is different, the distance between the characteristic peaks does not change, and remains at 2 mm, which is equal to the crack width *c*_1_; the positions of the characteristic peaks are basically the same, and they appear at the *x* coordinates of − 1 mm and 1 mm respectively, and do not appear different.

From the above phenomena, it can be seen that the HMII can be used as a characteristic quantity to detect the crack depth, although they are only nonlinear relationships, and the HMII cannot quantitatively evaluate the crack depth. As shown in Fig. 12, by comparing with the HMII change curve in the non-defect state, it can be judged whether the crack exists. If there is a characteristic peak in the change curve, there is a crack; otherwise, there is no crack. The relative size of the crack depth can be qualitatively compared by the size of the characteristic peak-to-peak value. As shown in Fig. 12, when the crack depth is small, the peak value of the characteristic wave peak is small; when the crack width is large, the peak value of the characteristic wave peak is relatively large. When the crack depth increases to a certain extent, the characteristic peak-to-peak value tends to be stable and does not increase any more. It is no longer possible to compare the relative size of the crack depth through the size of the characteristic peak-to-peak value.

The distance between the characteristic peaks and the position where the characteristic peaks appear remain unchanged, because the crack width is set to 2 mm at this time and remains unchanged. Through the previous analysis of the influence of the crack width on the MF distribution, we know that the peak distance of the characteristic peak and the position of the characteristic peak are related to the crack width, and the peak distance of the characteristic peak is equal to the crack width. Therefore, when the crack width is kept at 2 mm to study the effect of crack depth on the MF distribution, the distance between the characteristic peaks (characteristic peak distance) and the position of the characteristic peaks appear to remain unchanged at 2 mm.

**Figure 13:**
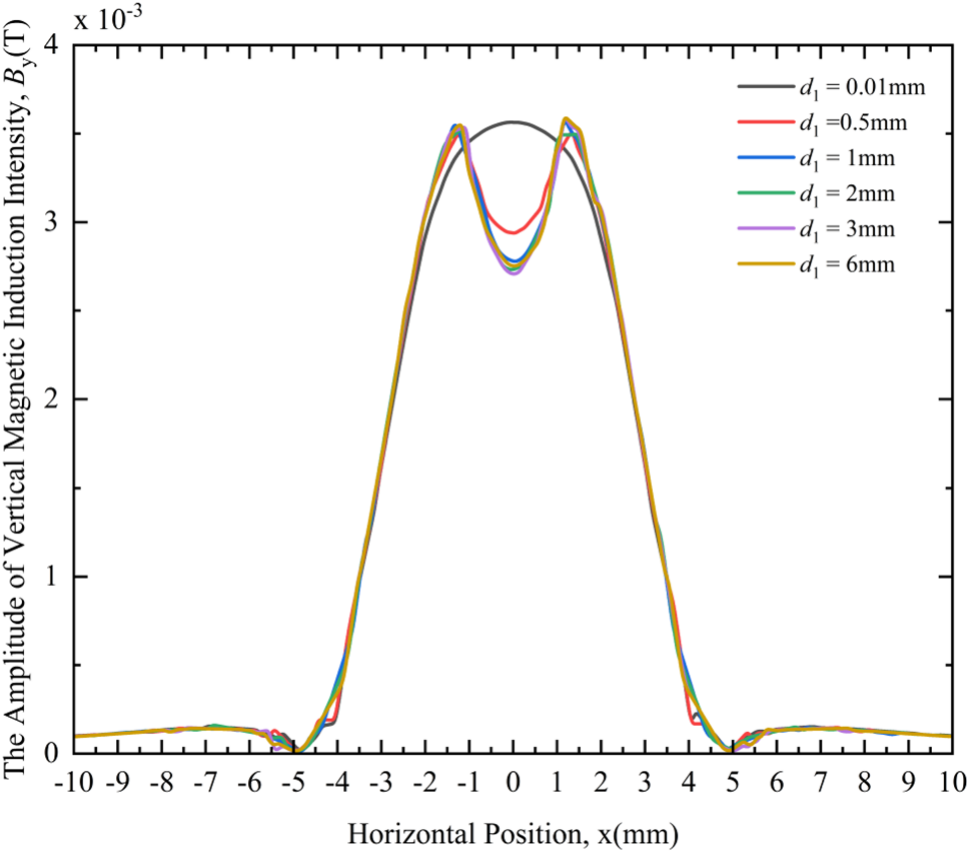
Variation curves of the VMII under different crack depths.

Figure 13 shows the variation law of the VMII amplitude under different crack depths. From the MF responses of different crack depths in Fig. 13, the following rules can be obtained:

1. Under different crack depths, the distribution of VMII is very different. When the crack depths are 0.5 mm, 1 mm, 2 mm, 3 mm, and 6 mm, the change curves of the HMII around the cracks are obviously different from the case where the crack depth is 0.01 mm (considered no defect), and the change curves will have a depression at the 0 coordinate.
2. When *d*_1_ = 0.01 mm, as the horizontal distance from the 0 coordinate increases, the VMII gradually decreases and tends to zero, after that, it gradually increases slightly and then gradually decreases again. The overall trend is "low on both sides, high in the middle", and a peak will appear at the 0 coordinate.
3. When *d*_1_ = 0.5 mm, 1 mm, 2 mm, 3 mm and 6 mm, as the crack depth gradually increases, the depression of VMII change curve at the 0 coordinate becomes deeper and deeper; When the crack depth increases to a certain extent (such as 1 mm), the depression no longer deepens with the increase of the crack depth, and begins to stabilize.

From the above phenomena, it can be seen that the existence of cracks can be qualitatively judged by comparing the VMII change curve with the state without defects. That is, if the change curve has a depression at the 0 coordinate, the crack exists; otherwise, the crack does not exist. The relative size of the crack depth can be qualitatively compared by the size of the degree of depression at the 0 coordinate, which is only applicable to the case of a small crack depth.

**Figure 14:**
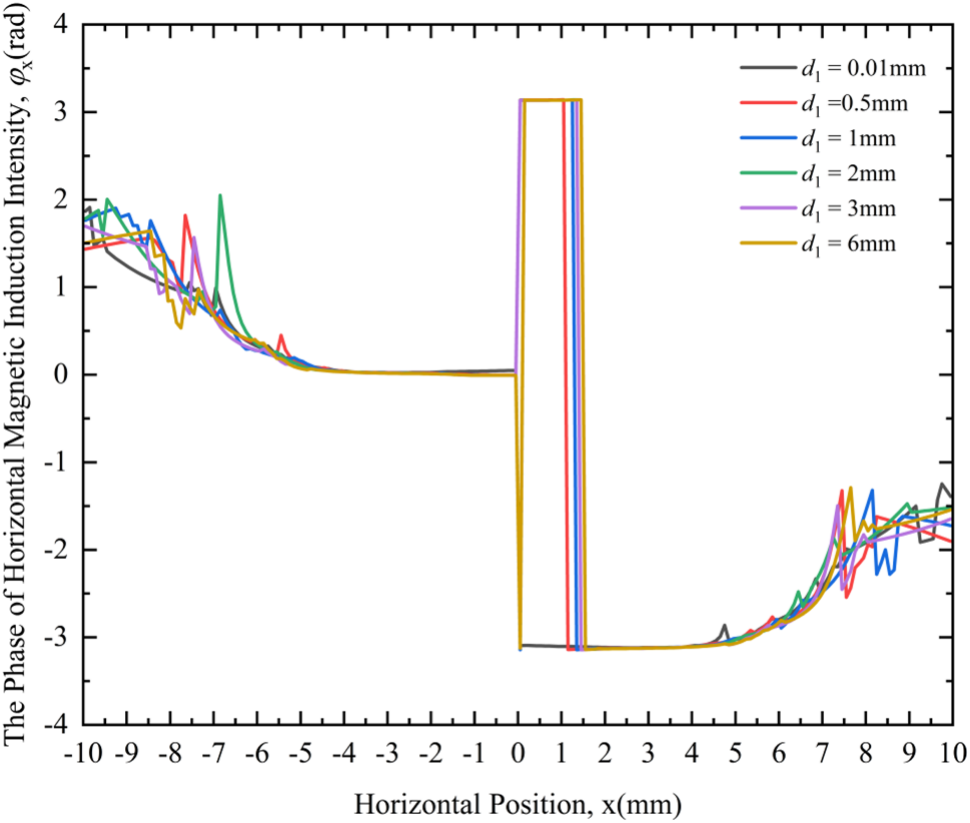
Variation curves of the PHMII under different crack depths.

Figure 14 shows the variation law of the PHMII under different crack depths. From the MF responses of different crack depths in Fig. 14, the following rules can be obtained:

Under different crack depths, the phase distribution of the HMII is very different. When the crack depth is 0.5 mm, 1 mm, 2 mm, 3 mm and 6 mm respectively, the change curve of the PHMII around the crack is obviously different from the case where the crack depth is 0.01 mm (considered no defect), and there will be a section where the phase remains π. When the crack width is different, the interval where the phase remains at π remains basically unchanged, and there is no expansion.

It can be seen from the above phenomena that the phase information of the HMII can be used to detect cracks. By comparing with the phase change curve of the HMII in the non-defect state, it can be qualitatively judged whether the crack defect exists. If there is a phase lag, there is a defect; otherwise, there is no defect. However, it is impossible to qualitatively deduce the depth of cracks by comparing the size of the interval where the phase is maintained at π like the different widths of cracks.

**Figure 15:**
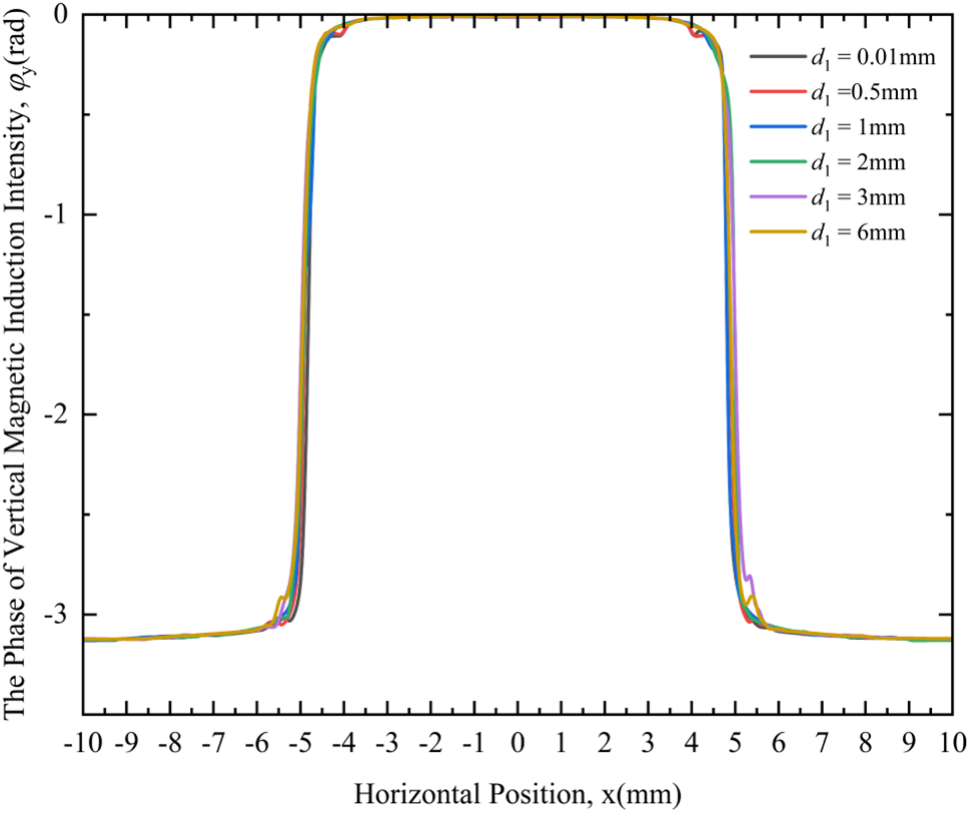
Variation curves of the PVMII under different crack depths.

Figure 15 shows the change law of the PVMII under different crack depths.

It can be seen from Fig. 15 that under different crack depths, the phase change curves of the VMII have very little difference and are difficult to distinguish. Similar to the crack width, the PVMII is also insensitive to the crack depth, so the PVMII cannot be used as a feature quantity for crack depth detection.

In this section, whether there is a characteristic peak at ± 1 mm on the horizontal magnetic induction variation curve, or a symmetric peak at a fixed position due to a depression in the center of the vertical magnetic induction variation curve, the reason is that the magnetic permeability of the air gap at the crack is significantly smaller than the relative magnetic permeability of the rotating shaft, a part of the magnetic field will bypass the crack and pass through the nearby objects, and the magnetic force lines will concentrate on both sides of the crack, resulting in a sharp increase in the magnetic induction here. The hysteresis phenomenon of the horizontal magnetic induction intensity change curve is ultimately due to the change of the relative permeability of the region caused by the crack, which leads to the distortion of the magnetic force line, and the magnetic force line flows to the area with smaller reluctance on both sides. Or, from the perspective of electromagnetic waves, the arrival time of electromagnetic waves reflected by cracks of different depths is inconsistent, which causes phase lag.

### Magnetic field distribution law of crack defect with different hidden depths

Keep the crack width *c*_1_ = 1 mm and crack depth *d*_1_ = 1 mm unchanged, and study its influence on the surface MF around the defect by changing the crack hidden depth. The influence of different crack hidden depths on the distribution of the MF mainly focuses on the amplitude of the HMII and VMII and their respective phases. The variation range of the crack hidden depth is *d*_2_ = 0.01 mm, *d*_2_ = 0.1 mm, *d*_2_ = 0.5 mm, *d*_2_ = 1 mm, *d*_2_ = 1.5 mm and *d*_2_ = 2 mm.

**Figure 16:**
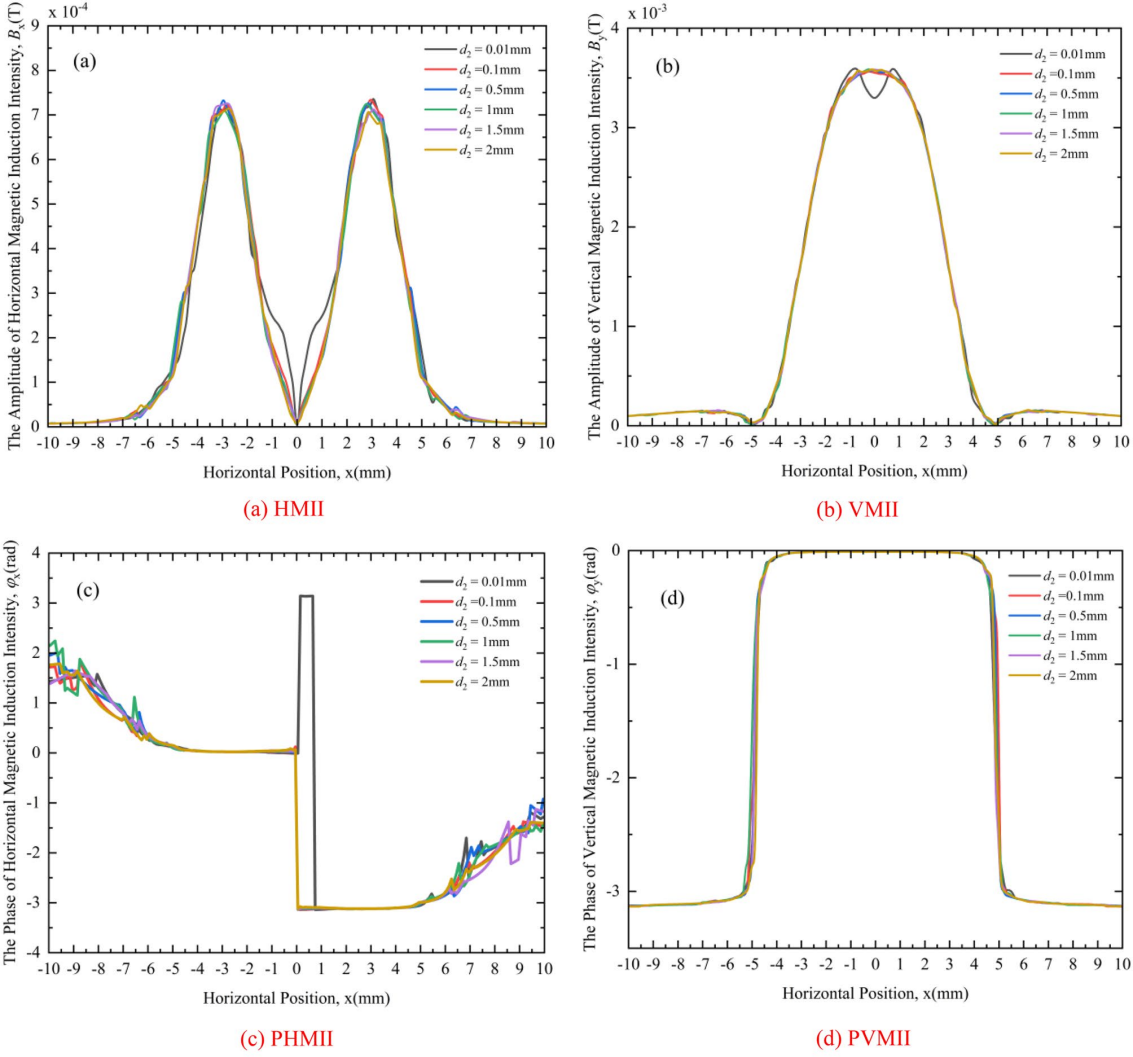
Change curves of MF under different crack hidden depths: (**a**) HMII, (**b**) VMII, (**c**) PHMII, (**d**) PVMII.

Under different crack hidden depths, the distributions of HMII, VMII, PHMII and PVMII are shown in Fig. 16a–d, respectively.

It can be seen from Fig. 16 that under different crack hidden depths, the change curves of HMII, VMII and PHMII are similar, that is: when the crack hidden depth (except 0.01 mm) is different, their change curves are quite different from those when the crack hidden depth is 0.01 mm (considering that there is no hidden crack), and this point can be used to judge whether there is a hidden crack. And because the hidden depth of cracks (except 0.01 mm) is different, their variation curves are difficult to distinguish from each other, so it is impossible to judge the relative size of the hidden depth of hidden cracks from these quantities. Regarding the PVMII, similar to the previous analysis, the MF is also insensitive to the hidden depth of cracks, and it is impossible to judge whether there are hidden cracks, nor to compare the relative size of the hidden depth of cracks.

In this section, the horizontal and vertical magnetic induction intensity change curves and vertical magnetic induction phase change curves can be used to judge whether there are hidden cracks, but the relative size of hidden cracks cannot be judged. The reason is the existence of the skin effect, which enables the surface defects of the rotating shaft to be detected to determine whether there are hidden cracks, and because the eddy current only penetrates a certain distance on the surface of the rotating shaft and concentrates on the surface of the rotating shaft, it is impossible to judge the relative size of hidden depth of hidden cracks.

### Experimental verification

Due to the large size and high cost of the complete traction motor shaft, there are not yet complete experimental conditions. Therefore, this section takes the case of cracks with different widths or depths on the surface of the shaft as an example to carry out experimental verification. The measured coil voltage is easier to obtain. If the voltage signal in the experiment is consistent with the simulation result, it can prove the accuracy of the simulation model. In the simulation model, the voltage signal and the magnetic field related quantities are different observations solved in the same simulation environment, so the correctness of the simulation model can verify the correctness of the research results related to the magnetic field. The accuracy of the magnetic field analysis can be indirectly verified by comparing the voltage signal in the experiment with the simulation.

**Figure 17:**
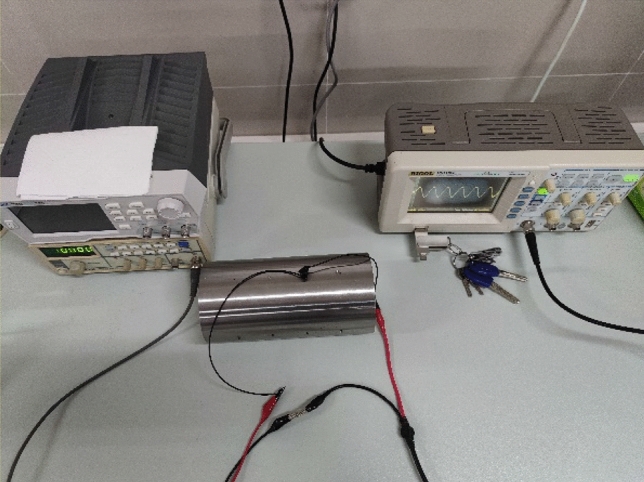
Eddy current testing experimental platform.

**Figure 18:**
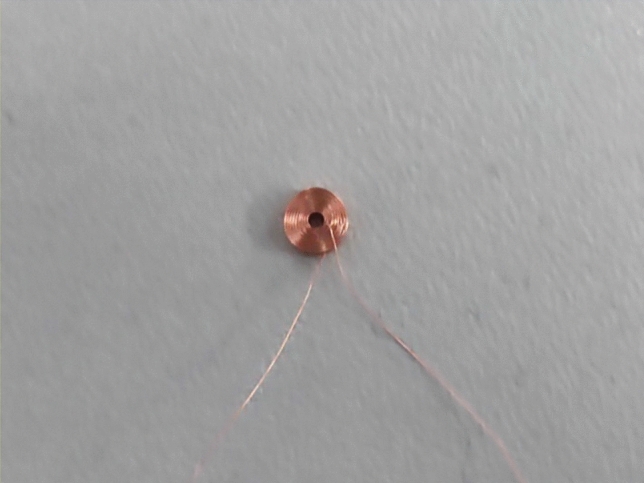
Coil physical map.

**Figure 19:**
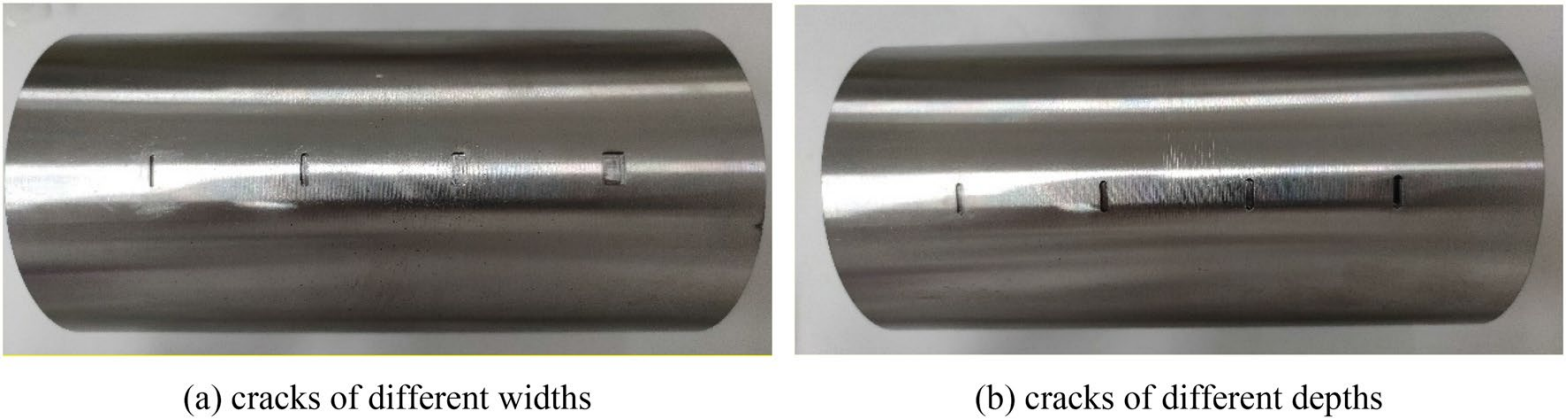
Rotary shafts to be tested with different defects on the surface.

The experimental platform is mainly composed of detection devices and test pieces, as shown in Fig. 17. The detection device mainly includes excitation source, coil, oscilloscope and so on. The actual coil is shown in Fig. 18, and the test piece is a rotating shaft with different defects in surface processing. There are two types of defects on the surface of the rotating shaft, as shown in Fig. 19, which are: crack defects with a width of 1 mm, 2 mm, 4 mm, and 6 mm; crack defects with a depth of 1 mm, 2 mm, 3 mm, and 6 mm.

During the experiment, all parameters were consistent with the simulation, and the coil moved on the surface of the rotating shaft and passed the defect position. Taking the position of the center of the defect as the 0 coordinate, the voltage signal changes in the coil are plotted and compared with the simulation data.

**Figure 20:**
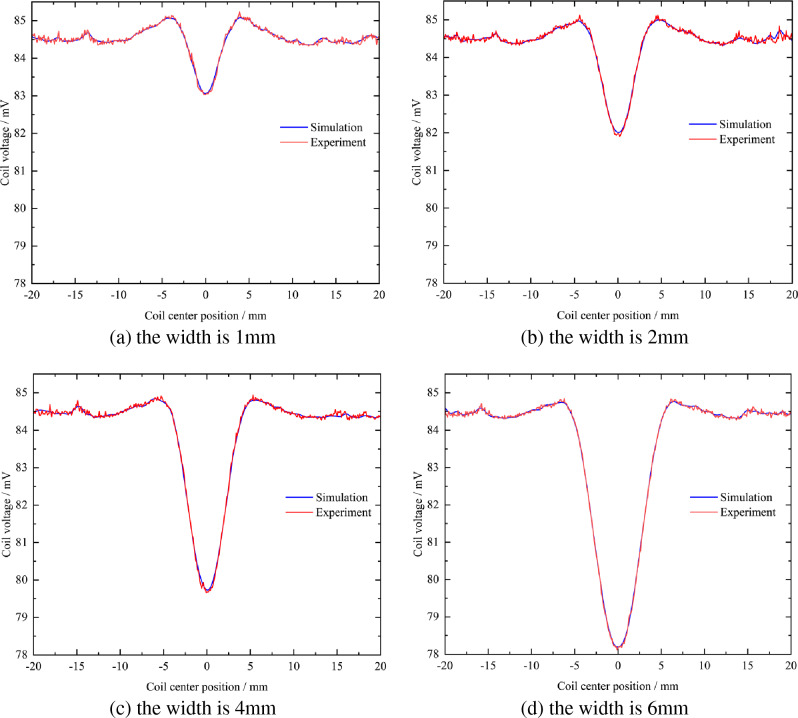
Comparison of voltage signals between simulation and experiment at different crack widths.

When the surface defects of the rotating shaft are cracks with a width of 1 mm, 2 mm, 4 mm, and 6 mm, the voltage signal changes in simulation and experiment are shown in Fig. 20.

It can be seen from Fig. 20a that when the crack width is 1 mm, the voltage signal change curves in the simulation and the experiment are roughly the same, and there will be depressions at the defects. The valley value of the depression is 83.03 mV in the simulation, and the valley value of the depression is about 83.00 mV in the experiment, basically keeping the same.

It can be seen from Fig. 20b that when the crack width is 2 mm, the voltage signal change curves in the simulation and the experiment are roughly the same, and depressions will appear at the defects. The valley value of the depression is 81.98 mV in the simulation, and the valley value of the depression is about 81.90 mV in the experiment, basically keeping the same.

It can be seen from Fig. 20c that when the crack width is 4 mm, the voltage signal change curves in the simulation and the experiment are roughly the same, and there will be depressions at the defects. The valley value of the depression is 79.68 mV in the simulation, and the valley value of the depression is about 79.60 mV in the experiment, basically keeping the same.

It can be seen from Fig. 20d that when the crack width is 6 mm, the voltage signal change curves in the simulation and the experiment are roughly the same, and there will be depressions at the defects. The valley value of the depression is 78.18 mV during the simulation, and the valley value of the depression is about 78.12 mV during the experiment, basically keeping the same.

**Figure 21:**
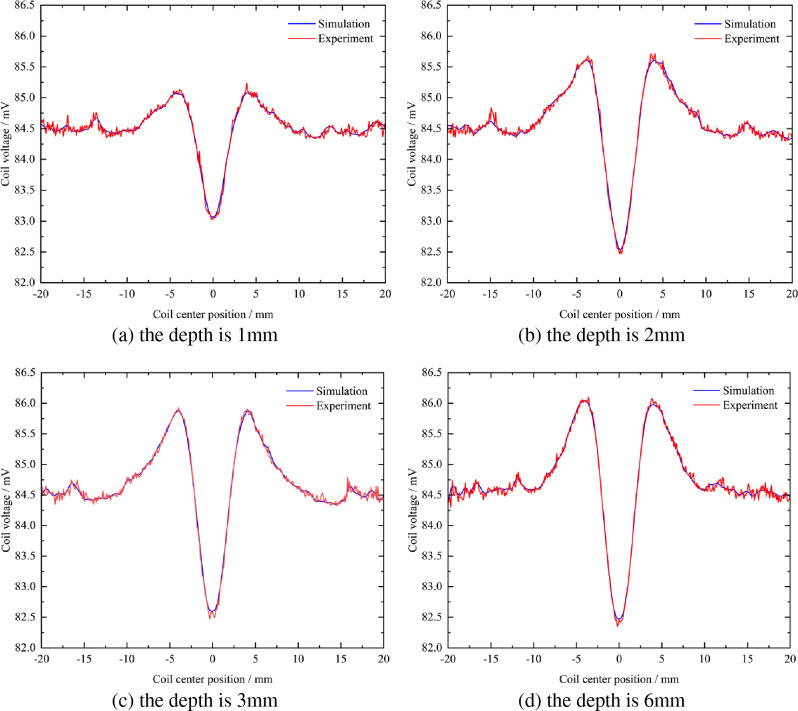
Comparison of voltage signals between simulation and experiment at different crack depths.

When the surface defects of the rotating shaft are cracks with a depth of 1 mm, 2 mm, 3 mm, and 6 mm, the voltage signal changes in simulation and experiment are shown in Fig. 21.

It can be seen from Fig. 21a that when the crack depth is 1 mm, the voltage signal change curves in the simulation and the experiment are roughly the same, and there will be depressions at the defects. The valley value of the depression is 83.03 mV in the simulation, and the valley value of the depression is about 83.00 mV in the experiment, basically keeping the same.

It can be seen from Fig. 21 that when the crack depth is 2 mm, the voltage signal variation curves in the simulation and the experiment are roughly the same, and depressions will appear at the defects. The valley value of the depression is 82.46 mV in the simulation, and the valley value of the depression is about 82.40 mV in the experiment, basically keeping the same.

It can be seen from Fig. 21c that when the crack depth is 3 mm, the voltage signal change curves in the simulation and the experiment are roughly the same, and there will be depressions at the defects. The trough value at the depression is 82.59 mV in the simulation, and about 82.50 mV in the experiment, basically keeping the same.

It can be seen from Fig. 21d that when the crack depth is 6 mm, the voltage signal change curves in the simulation and the experiment are roughly the same, and there will be depressions at the defects. The valley value of the depression is 82.45 mV in the simulation, and the valley value of the depression is about 82.35 mV in the experiment, basically keeping the same.

To sum up, the simulation and experiment voltage changes under different types of defects are basically consistent, which verifies the accuracy of the model and indirectly verifies the correctness of the magnetic field correlation analysis.

## Conclusion

In this paper, the distribution of the HMII, VMII, PHMII and PVMII near the crack on the surface of the rotating shaft under different parameters of the crack (width, depth and hidden depth) are obtained through simulation. After analyzing these distribution rules, the following results are obtained:

1. The parameters related to the magnetic field on the surface of the rotating shaft can be used to qualitatively determine whether there are cracks and to quantitatively evaluate the crack width. HMII, VMII and PHMII can be used to qualitatively and quantitatively evaluate crack width. PVMII cannot qualitatively and quantitatively evaluate crack width.
2. The parameters related to the magnetic field on the surface of the rotating shaft can qualitatively judge whether there is a crack or not and evaluate the relative size of the crack depth. HMII, VMII and PHMII can be used to qualitatively evaluate the crack depth. PVMII cannot qualitatively and quantitatively evaluate crack depth.
3. The parameters related to the MF on the surface of the rotating shaft can qualitatively determine whether there are hidden cracks. In the change curves of HMII, VMII and PHMII, only whether there are hidden cracks can be judged; the PVMII is still insensitive to the crack hidden depth.

## Data Availability

The datasets generated and analyzed during the current study are not publicly available due the data also forms part of an ongoing study, but are available from the corresponding author on reasonable request.
